# Inactivation of the Prelimbic Cortex Impairs the Context-Induced Reinstatement of Ethanol Seeking

**DOI:** 10.3389/fphar.2017.00725

**Published:** 2017-10-17

**Authors:** Paola Palombo, Rodrigo M. Leao, Paula C. Bianchi, Paulo E. C. de Oliveira, Cleopatra da Silva Planeta, Fábio C. Cruz

**Affiliations:** ^1^Laboratory of Pharmacology, School of Pharmaceutical Sciences, Universidade Estadual Paulista, São Paulo, Brazil; ^2^Joint Graduate Program in Physiological Sciences UFSCar/UNESP, São Carlos, Brazil; ^3^Departamento de Biorregulação, Instituto de Ciências da Saúde, Universidade Federal da Bahia, Salvador, Brazil; ^4^Department of Pharmacology, São Paulo Federal University, São Paulo, Brazil

**Keywords:** prelimbic, pharmacologic inactivation, context, reinstatement, ethanol

## Abstract

Evidence indicates that drug relapse in humans is often provoked by exposure to the self-administered drug-associated context. An animal model called “ABA renewal procedure” has been used to study the context-induced relapse to drug seeking. Here, we reported a new and feasible training procedure for the ABA renewal method to explore the role of the prelimbic cortex in context-induced relapse to ethanol seeking. By using a saccharin fading technique, we trained rats to self-administer ethanol (10%). The drug delivery was paired with a discrete tone-light cue. Lever pressing was subsequently extinguished in a non-drug-associated context in the presence of the discrete cue. Rats were subsequently tested for reinstatement in contexts A or B, under extinction conditions. Ethanol-associated context induced the reinstatement of ethanol seeking and increased the expression of Fos in the prelimbic cortex. The rate of neural activation in the prelimbic cortex was 3.4% in the extinction context B and 7.7% in the drug-associated context A, as evidenced by double-labeling of Fos and the neuron-specific protein NeuN. The reversible inactivation of the neural activity in the prelimbic cortex with gamma-Aminobutyric acid (GABA) receptor agonists (muscimol + baclofen) attenuated the context-induced reinstatement of ethanol self-administration. These results demonstrated that the neuronal activation of the prelimbic cortex is involved in the context-induced reinstatement of ethanol seeking.

## Introduction

Ethanol is the most commonly used addictive substance worldwide (UNODC, 2016). The harmful use of ethanol is responsible for 3.3 million deaths each year (UNODC, 2016). Relapse represents a prevalent and significant problem in ethanol addiction. Indeed, given the high rate of recidivism in alcoholism, relapse is clearly a major impediment to treatment efforts of this disorder (Fox et al., 2008; Sinha, 2009; Becker and Koob, 2016).

Evidence indicates that drug relapse in humans is often provoked by exposure to the self-administered drug-associated context (O’Brien et al., 1990; Gauggel et al., 2010). In this regard, clinical reports and laboratory studies have shown that factors associated with ethanol consumption may induce relapse in humans (O’Brien et al., 1990; Kirk and de Wit, 2000; Litt and Cooney, 2000). For instance, abstinent alcoholics have reported that specific environmental cues elicit craving for ethanol (McCusker and Brown, 1990; Litt et al., 2000; Gauggel et al., 2010). An animal model known as “ABA renewal procedure” has been used to study the context-induced relapse to drug seeking (Bouton et al., 2006; Crombag et al., 2008; Bouton, 2011, 2014; Bouton et al., 2014; Bouton and Schepers, 2015). In this animal model, rats are trained to self-administer the drug in a context (context A); during the training, drug infusions are paired with a discrete tone-light cue. The lever pressing is subsequently extinguished in a non-drug-associated context (context B) in the presence of the discrete cue. The rats are subsequently tested for reinstatement in the drug-associated context under extinction conditions (Crombag et al., 2008; Lasseter et al., 2010; Marchant et al., 2013). Since it was first demonstrated by Burattini et al. (2006), context-induced ethanol seeking has been replicated in several studies (Bossert et al., 2013; Marchant et al., 2014; Willcocks and McNally, 2014).

Studies have demonstrated the participation of cortical areas in both relapse and extinction of drug seeking (Bossert et al., 2012; Willcocks and McNally, 2013; Marchant et al., 2015). Specifically, the prelimbic cortex is associated with reinstatement of drug seeking, while the infralimbic cortex is implicated in the extinction of drug seeking (Peters et al., 2009; Van den Oever et al., 2010). In this regard, it was found that inactivation of the prelimbic cortex (using the sodium channel blocker tetrodotoxin-TTX) impaired the context-induced cocaine seeking (Fuchs et al., 2005). Moreover, the reversible inactivation (using muscimol + baclofen, gamma-aminobutyric acid (GABA) a + GABAb agonists, respectively) of the infralimbic cortex impaired the extinction of cocaine self-administration (Muigg et al., 2008). Similarly, Willcocks and McNally (2013) reported that the reversible inactivation of prelimbic cortex by muscimol + baclofen decreases context-induced reinstatement of alcoholic beer-seeking.

However, the modulation of extinction and seeking behavior by infralimbic *versus* prelimbic cortex is supported by numerous studies (Rogers et al., 2008; Koya et al., 2009a; Rocha and Kalivas, 2010; Warren et al., 2016). While some studies reported a role of the prelimbic cortex in drug seeking behavior (McFarland and Kalivas, 2001; Kalivas and McFarland, 2003; Peters et al., 2009; Calu et al., 2013), other studies have indicated that the inactivation of the prelimbic areas does not attenuate the reinstatement of drug seeking (Koya et al., 2009a; Bossert et al., 2011; Li et al., 2015a). Although the infralimbic cortex has been implicated in the extinction of drug seeking (Peters et al., 2009; Warren et al., 2016), many studies have demonstrated that the inactivation of the ventral medial prefrontal cortex (brain area that comprises the infralimbic cortex) has no effect on this behavior (Rogers et al., 2008; Koya et al., 2009a; Rocha and Kalivas, 2010; Warren et al., 2016). Recently, it was demonstrated that the ventral medial prefrontal cortex encodes neuronal ensembles related to both food reward and extinction memories (Rhodes and Killcross, 2004, 2007; Ishikawa et al., 2008; Peters et al., 2008; Warren et al., 2016).

Here, we used Fos immunohistochemistry and the mixture of muscimol and baclofen in the activation procedure to examine whether the context-induced reinstatement of ethanol seeking is mediated by the prelimbic cortex.

## Materials and Methods

### Animals

We used rats (male Long-Evans, 350–450 g; *n* = 86), obtained from the animal breeding facility of the São Paulo State University-UNESP. Groups of four animals were housed in plastic cages (32 × 40 × 16 cm) with unrestricted access to food and water. Rats were continuously maintained on a reversed light/dark cycle (12 h/12 h, lights off at 07:00 a.m.) in a room with controlled temperature (23 ± 2°C). All experiments were performed during the dark phase. The experimental protocol was approved by the Ethical Committee for Use of Animal of Physical Institute of São Carlos, São Paulo University (Protocol #01/2015) and were conducted according to the ethics principles of the Conselho Nacional de Controle de Experimentação Animal (CONCEA).

Twenty-one rats were excluded from the study: 14 for poor training (<10 reinforcements/day) or because they did not reach the extinction criterion (<25 responses/day), four for misplaced cannulae, and three because they lost their head caps.

### Apparatus

Standard Med Associates (St. Albans, VT, United States) self-administration chambers were used in all experiments. Two different contexts were set up as described in Cruz et al. (2014). Context A corresponded to the ethanol paired context and context B corresponded to the non-drug extinction context.

### Drugs

The following compounds were used: Ethanol 96% (Synth); flunixine-meglumine (Schering-Plough); streptomycin and penicillin polyantibiotic (Fort Dodge); tribromoethanol (Sigma–Aldrich); baclofen (Tocris Bioscience); muscimol (Tocris Bioscience); and saccharin (Sigma).

### Experimental Design

**FIGURE 1:**
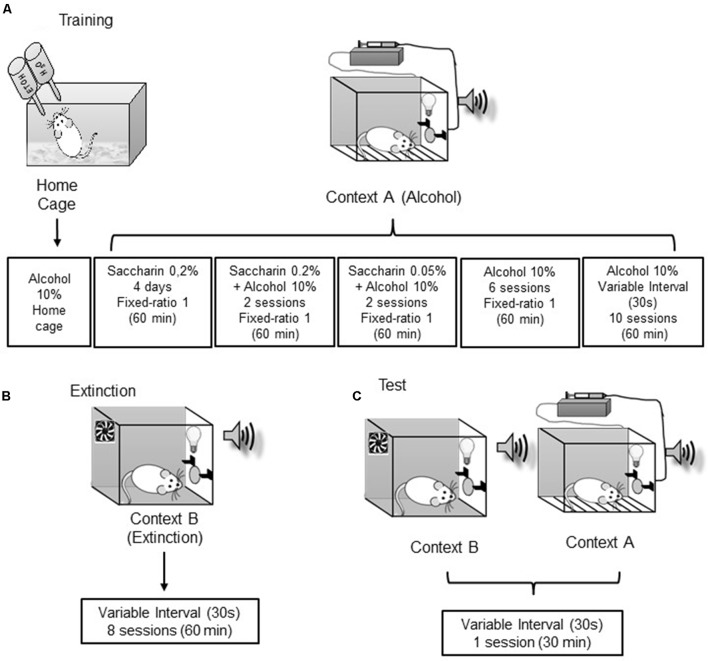
*Schematic representation of the experimental design*. The protocol was adapted from Willcocks and McNally (2013). Briefly, rats were given access to 10% ethanol in their home cages. Using a saccharin fading technique, rats are trained to self-administer 10% ethanol in context A. Subsequently, the lever pressing in the presence of the discrete cue was extinguished in context B. The context-induced reinstatement of drug seeking was assessed by re-exposing the rats to context A or B under extinction conditions. See Methods for more details. **(A)** Training phase; **(B)** Extinction phase; **(C)** Test day.

We used a protocol adapted from Willcocks and McNally (2013). By using a saccharin fading technique, we trained rats to self-administer ethanol (10%) over 24 days. The drug delivery was paired with a discrete tone-light cue. Lever pressing was subsequently extinguished in a non-drug-associated context in the presence of the discrete cue during 8 days. The rats were subsequently tested for reinstatement in context A or context B under extinction conditions (**Figure 1**).

#### Ethanol Self-administration Training

Initially, the rats had free access to ethanol (10% w/v) and water for 3 days consecutively in their homecages, to habituate them to the ethanol taste, as previously described by Leão et al. (2015). Subsequently, they were trained to self-administrate 10% ethanol using a saccharin fading procedure. Twenty-four sessions were performed during the training phase: Four sessions where 0.1 ml of 0.2% saccharin solution was delivered, followed for two sessions where an active lever press resulted in the delivery of 0.1 ml of 0.2% saccharin-plus-10% ethanol (w/v). Subsequently, we performed two sessions where an active lever press resulted in the delivery of 0.1 ml of 0.05% saccharin-plus-10% ethanol (w/v), followed by six sessions where only 10% ethanol (w/v) was delivered. During these phases of the training in context A, saccharin and/or ethanol reinforcements were earned under a fixed ratio 1 (FR1) with a 20-s timeout reinforcement schedule and paired with a compound tone (2,900 Hz; 20 dB above background) and light (7.5 W white light) cue for 2.3 s. Following the FR1 session, we trained rats on a variable-interval 30-s (VI-30) schedule of reinforcement for 10 sessions (Marchant et al., 2016). During the VI-30 sessions, a 10% ethanol (w/v) delivery was available after an active lever press at pseudo-random intervals (1–59 s) after the preceding ethanol delivery. The ethanol deliveries were also paired with a compound tone (2,900 Hz; 20 dB above background) and light (7.5 W white light) cue for 2.3 s.

The initiation of each training session was signaled by the illumination of the house-light and the insertion of the active lever into the chamber. Inactive lever presses had no programmed consequences. All training sessions were performed for 1 h each.

After the end of the last VI-30 session, blood samples (50 μl) were collected from the tip of the tail of each rat. The blood samples were analyzed by an enzymatic system (AM1 Analyzer, Analox Instruments Ltd, London, United Kingdom) on the basis of the measurement of oxygen consumption in the ethanol-acetaldehyde reaction. This procedure was performed to check if all the delivered ethanol was consumed by the animals.

#### Extinction of Ethanol Self-administration

Extinction was performed in a non-drug-associated context (context B) in the presence of the same discrete cue described above, but the responses on the previously active lever were reinforced by ethanol delivery. The ethanol self-administration behavior was considered extinct when the rats met the extinction criterion of <15 presses per 1 h session. A minimum of eight extinction sessions were performed.

#### Test for Context-Induced Reinstatement

We tested the rats for ethanol seeking (active lever presses under extinction conditions) in 30-min sessions in contexts A or B under extinction parameters (VI-30 schedule of reinforcement), in which an active lever press was not reinforced by ethanol delivery (Cruz et al., 2014).

### Implantation of Intracranial Cannulas

After the rats were anesthetized with tribromoethanol (250 mg/kg; intraperitoneal injection [i.p.]), permanent guide cannulas (23-gauge, Master-One Ribeirão Preto, São Paulo, Brazil) were implanted bilaterally 1 mm above the prelimbic cortex. We used the stereotaxic coordinates according to Paxinos and Watson (2005) and according to Willcocks and McNally (2013). The nose bar was set at -3.3 mm and the coordinates for the prelimbic cortex were as follows: anteroposterior (AP) +3.0 mm, medial lateral (ML) ± 1.5 mm (10° angle), and dorsal ventral (DV) -3.0 mm (**Figure 5C**). After the surgery, the rats were treated with a streptomycin and penicillin polyantibiotic formulation (0.27 mg/kg, intramuscular injection [i.m.]; Pentabiotico, Fort Dodge, Campinas, São Paulo, Brazil) to prevent infections, and received the non-steroidal anti-inflammatory drug flunixine-meglumine (0.025 mg/kg, i.m.; Banamine, Schering-Plough, Cotia, São Paulo, Brazil) for post-operative analgesia.

### Intracranial Injections and Histology

Fifteen minutes prior to the test, bilateral injections of saline or muscimol (0.03 nmol/0.5 μl/side) + baclofen (0.3 nmol/0.5 μl/side) (Tocris Bioscience) dissolved in saline were performed in the prelimbic cortex. The doses were based on previous studies (McFarland and Kalivas, 2001; Bossert et al., 2011; Cruz et al., 2014). The intracranial injections were administered using a syringe pump (Harvard Apparatus, Holliston, MA, United States) and 10 μl Hamilton syringes that were attached via polyethylene 50 tubing to 30-gauge injectors (Plastics One). Muscimol + Baclofen or saline were injected over 1 min and the injectors were left in place for 1 min. At the end of the study, the rats were injected with an overdose of tribromoethanol (500 mg/kg, i.p.). Subsequently, their brains were removed, frozen, and sectioned coronally at 40 μm using a cryostat. All sections containing the cannula tracts were collected, stained for cresyl violet, and coverslipped with Permount (Sigma).

### Experiments

#### Experiment 1: Context-Induced Reinstatement of Ethanol Seeking

In the test group (A-B-A), ethanol self-administration was trained in context A, extinction training in context B (1 h per day), and reinstatement test in context A (30 min). In the control group (A-B-B), ethanol self-administration was trained in context A, extinction training in context B (1 h per day), and reinstatement test in context B (30 min). A total of 34 animals were used. The experimental design was based on Cruz et al. (2014).

#### Experiment 2: Prelimbic Cortex Neuronal Activation after Context-Induced Reinstatement of Ethanol Seeking

We used immunohistochemistry to characterize the involvement of the prelimbic cortex in the context-induced reinstatement of ethanol operant self-administration.

At the end of the reinstatement test, the rats used in Experiment 1, were anesthetized with tribromoethanol (250 mg/kg, i.p.) and perfused with 100 ml phosphate-buffered saline (PBS) followed by 400 ml 4% paraformaldehyde.

The brains were post-fixed in 4% paraformaldehyde for 90 min and transferred to 30% sucrose in PBS at 4°C for 2–3 days. Brains were frozen in powdered dry ice and kept at -80°C until sectioning. Coronal sections were cut at 40 μm. Free-floating sections were washed three times in PBS, blocked with 3% normal goat serum (NGS) in PBS with 0.25% Triton X-100 (PBS-Tx), and incubated for 24 h at 4°C with anti-Fos antibody (1:4000, sc-52; Santa Cruz Biotechnology) diluted in blocking solution. After further washing with PBS, sections were incubated for 2 h with biotinylated goat anti-rabbit secondary antibody (1:400; Vector Laboratories) in PBS-Tx and 1% NGS. After washing in PBS, sections were incubated for 1 h in avidin-biotin-peroxidase complex (ABC Elite kit, PK-6100; Vector Laboratories) in PBS containing 0.5% Triton X-100. Finally, sections were washed in PBS and developed in 3,3′-diaminobenzidine for approximately 3 min, transferred into PBS, and mounted onto chrome alum-gelatin-coated slides. Once dry, the slides were dehydrated through a graded series of alcohol and cleared with xylol (LabSynth, SP, Brazil) before coverslipping with Permount (Sigma-Aldrich, St. Louis, MA, United States).

Bright-field images of Fos immunoreactivity in the prelimbic cortex were captured by using a CCD camera (Coolsnap Photometrics, Roper Scientific Inc.) and QimagingExi Aqua attached to a Zeiss Axioskop 2 microscope. Images for counting the labeled cells were captured at 100 × magnification. Labeled cells from 3–4 hemispheres per rat were automatically counted using IPLab software for Macintosh, version 3.9.4 r5 (Scanalytics Inc.) and iVision for Macintosh, version 4.0.15 (Biovision). Each rat was considered as one sample, and the cell counts from all the images of each rat were averaged for statistical comparisons (Cruz et al., 2014).

#### Experiment 3: Quantification of the Activated Cortex Neurons Using Double-Labeling Immunofluorescence

We used double-labeling immunofluorescence to characterize the neuronal activation of the prelimbic cortex during the reinstatement tests. For these experiments, we used four animals from each group. All rats were perfused with 4% paraformaldehyde 90 min after the beginning of the reinstatement test. The dissected brains were processed as described above and were kept at -80°C until sectioning. Coronal sections were cut between bregma +2.5 mm and +3.7 mm (Paxinos and Watson, 2005).

For these assays, we used sections obtained from a subset of the brains used in the previous Fos immunohistochemistry assays (*n* = 4 rats from each group). The proportion of all prelimbic cortical neurons expressing Fos during the reinstatement test were determined by double-labeling for Fos and the neuron-specific protein NeuN. For Fos + NeuN labeling, sections were washed three times in Tris-buffered saline (TBS) and permeabilized for 30 min in TBS with 0.2% Triton X-100. Sections were incubated in primary antibodies diluted in TBS with 0.2% Triton X-100 for 24 h on a shaker at 4°C. Primary antibodies were rabbit anti-Fos (1:400, sc-52; Santa Cruz) and mouse anti-NeuN (1:2000, MAB37; EMD Millipore). The sections were further washed three times in TBS and incubated with secondary fluorescent antibodies diluted in TBS with 0.2% Triton X-100 for 2 h on a shaker at room temperature. The secondary antibodies were Alexa Fluor 488-labeled donkey anti-rabbit (1:200, A-10042; Invitrogen) and Alexa Fluor 560 donkey anti-mouse (1:2000, A-10238; Invitrogen) to label Fos and NeuN, respectively. After labeling, sections were washed in TBS, mounted onto chrome alum-gelatin-coated slides, and coverslipped with VectaShield hard-set mounting media. All fluorescent images of the prelimbic cortex were captured by using a CCD camera (Coolsnap Photometrics, Roper Scientific) attached to a Zeiss Axioskop 2 microscope. Images for the co-localization of Fos and NeuN were captured at 200× magnification. The number of Fos-labeled and double-labeled cells from the prelimbic cortex of one section per rat were counted using iVision for Macintosh, version 4.0.15 (Biovision Technologies).

We determined the proportion of all prelimbic cortical neurons expressing Fos (i.e., Fos^+^NeuN^+^ cells) during the reinstatement test as described in Cruz et al. (2014).

#### Experiment 4: Effect of the Pharmacological Inactivation of the Prelimbic Cortex on the Context-Induced Reinstatement of Ethanol Seeking

Thirty-one Long-Evans rats were anesthetized and implanted with permanent bilateral guide cannulas into the prelimbic cortex as described above. Subsequently, rats underwent ethanol self-administration training and extinction as described above. On the test day, the rats received bilateral injections of either muscimol (0.03 nmol/0.5 μl/side) + baclofen (0.3 nmol/0.5 μl/side) (TocrisBioscience) dissolved in sterile saline 0.9% or saline 0.9% alone, 15 min prior to the reinstatement test as described above. The number of rats per group was as follows: vehicle context B, *n* = 8; baclofen + muscimol context B, *n* = 6, vehicle context A, *n* = 9; and baclofen + muscimol context A, *n* = 8. To rule out the possibility that the effect of baclofen + muscimol on the test day was due to motor deficits, 18 rats were trained after the completion of this experiment to lever-press for 0.2% of saccharin under an FR1 and 20-s timeout reinforcement schedule for five 60-min sessions. Subsequently, we assessed the effect of vehicle or baclofen + muscimol injections into the prelimbic cortex on the saccharin-maintained responding in a 30-min session. At the end of the test session, the rats were deeply anesthetized with tribromoethanol (500 mg/kg) and perfused with 100 ml PBS followed by 400 ml paraformaldehyde (4%). The brains were post-fixed in 4% paraformaldehyde for 90 min and transferred to 30% sucrose in PBS at 4°C for 2–3 days. The brains were frozen in powdered dry ice and kept at -80°C until sectioning.

### Statistical Analyses

All statistical analyses were performed by using Statistic, StatSoft. The data were analyzed by analysis of variance (ANOVA); Newman–Keuls test was used for *post hoc* analyses when the ANOVA indicated significant main or interaction effects (*p* < 0.05).

## Results

### Experiments 1–4: Training and Extinction

**FIGURE 2:**
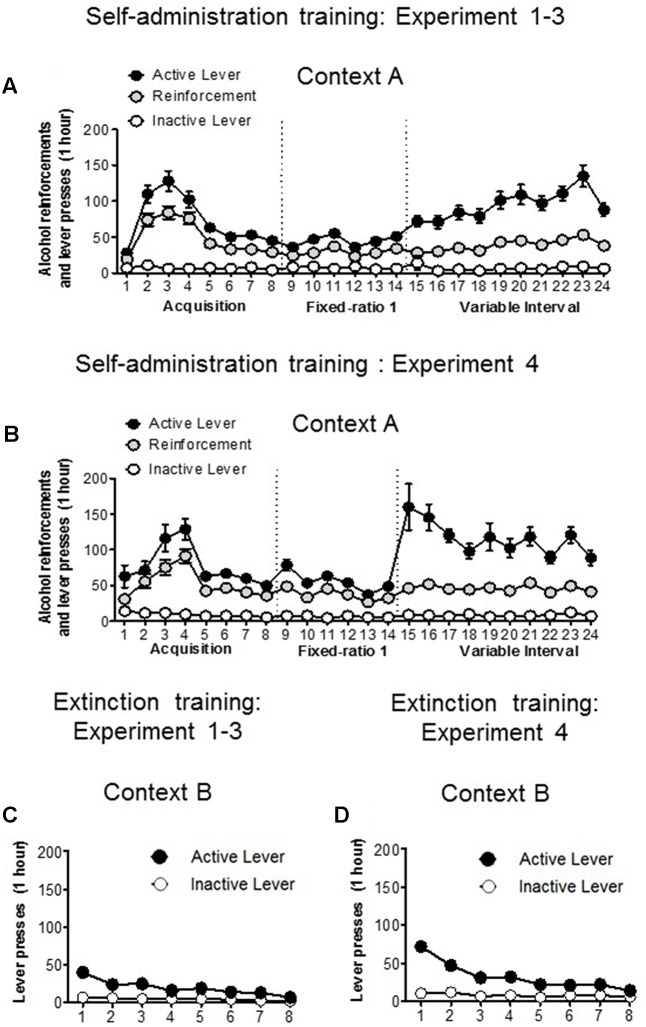
Training and extinction of ethanol reinforcement responding. **(A,B)** Rats were trained to self-administer ethanol over 24 days. Data depict the mean (±standard error of the mean [SEM]) number of infusions, and active and inactive lever presses during ethanol self-administration training in context A in Experiments 1–3 (*n* = 34) and experiment 4 (*n* = 31). **(C,D)** Mean (±SEM) number of presses on the active lever and inactive lever during eight extinction sessions conducted in context B in Experiments 1–4.

**FIGURE 3:**
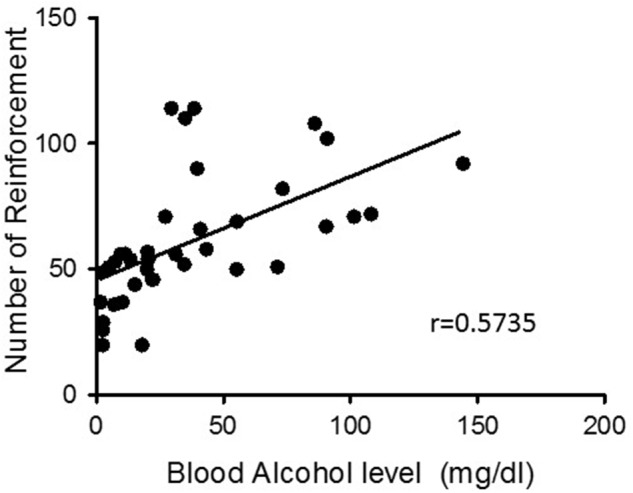
Blood ethanol determination. Immediately after the end of the last VI-30 session, blood samples (50 μl) were collected from the tip of the tail of each rat. The graph depicts the correlation plots of the number of reinforcements achieved during the last training session as a function of the blood ethanol level (*p* < 0.01).

**Figures 2A,B** depicts the mean (±standard error of the mean [SEM]) the number of ethanol reinforcements and presses on the active and inactive levers during the training phase in context A in all experiments. The rats displayed a consistent ethanol self-administration, as indicated by the increase in the number of infusions and active lever presses over the training sessions. **Figures 2C,D** depicts the mean (±SEM) number of lever presses on the previously active and inactive levers during the first eight extinction sessions in context B. As expected, the active lever presses decreased over time. **Figure 3** shows the correlation between the number of reinforcements achieved during the last training session and the blood ethanol levels, indicating a significant correlation (*r* = 0.5735, *p* < 0.01; **Figure 3**).

**FIGURE 4:**
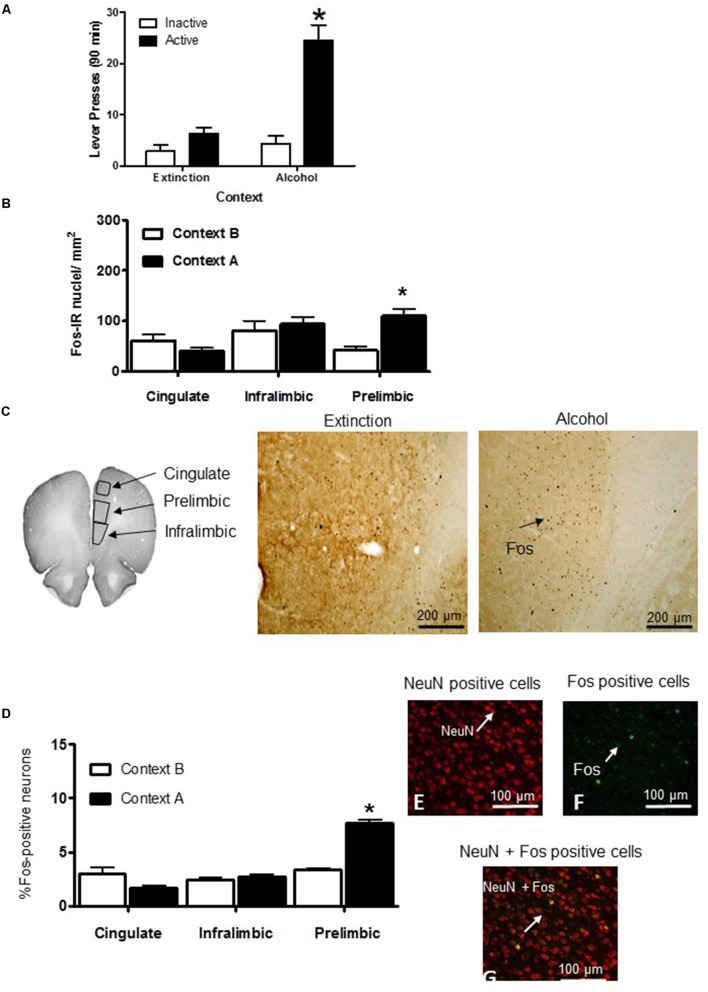
Context-induced reinstatement of ethanol seeking increased the Fos expression in the prelimbic cortex. Data depict the mean (±standard error of the mean). **(A)** Test: Total number of active lever and inactive lever presses in rats tested in contexts A and B. ^∗^Different from context B, *p* < 0.05, *n* = 9–17 per group. **(B)** Number of Fos-immunoreactive (IR) nuclei per mm^2^ in the cingulate, infralimbic, and prelimbic cortex. **(C)** Areas used for quantifying the Fos-IR neurons in the cingulate, infralimbic, and prelimbic cortex of rats. ^∗^Different from context B, *p* < 0.05 (*n* = 9–17 per group). **(D)** Percentage of neural activation by double-labeling with Fos and NeuN [(Fos^+^/NeuN^+^cells) × 100] per mm^2^ in the cingulate, infralimbic, and prelimbic cortex. **(E)** Red: neuronal nuclei marker NeuN. **(F)** Green: Fos expression. **(G)** Yellow: Fos + NeuN double-labeled nuclei. Scale bar = 100 μm, *n* = 4 per group.

For experiments 1–3, on the test day, we assessed the context-induced reinstatement of ethanol seeking by assessing the non-reinforced lever presses in context A *versus* context B (**Figure 4A**). The exposure to context A, but not context B, increased the non-reinforced active lever pressing. The results indicated a significant interaction between context (A and B) and lever (active and inactive) (*F*_1,64_ = 19.89, *p* < 0.0001).

### Experiment 2: Context-Induced Reinstatement of Ethanol Seeking Is Associated with Increased Fos Expression in the Prelimbic Cortex, But Not in the Infralimbic and Cingulate Cortex

In Experiment 2, we determined whether the context-induced reinstatement of ethanol seeking was associated with increased Fos-immunoreactive nuclei (Fos-IR) in the prelimbic, infralimbic, and cingulate cortex (**Figure 4C**). We analyzed the Fos expression by using the between-subject factor of context (contexts A and B) and the within-subject factor of the cortical region (cingulate, infralimbic, and prelimbic).

Exposure to context A increased the number of Fos-IR nuclei in the prelimbic cortex, but not in the infralimbic and cingulate cortical regions (**Figure 4B**). The results in the prelimbic cortex further demonstrated a significant main effect of the context (*F*_1,17_ = 6.24; *p* < 0.01). The number of Fos-IR nuclei was higher in context A than in context B (*p* < 0.01).

#### Experiment 3: Quantification of Activated Cortex Neurons Using Double-Labeling Immunofluorescence

In Experiment 3, we used double-labeling immunofluorescence (**Figures 4E–G**) to determine the percentage of Fos-expressing neurons in sections obtained from a subset of the brains used in Experiment 1 (*n* = 4 rats per group). In the prelimbic cortex, the percentage of activated neurons was 3.4 ± 0.1% and 7.74 ± 0.3% of all neurons following the exposure to context B and context A, respectively. The percentage of activated neurons observed in the infralimbic cortex was 2.5 ± 1.3% and 3.16 ± 0.62% following the exposure to context B and context A, respectively. The percentage of activated neurons in the cingulated cortex was 1.1 ± 0.1% and 3.96 ± 1.31% following exposure to contexts B and A, respectively (**Figure 4D**). The results in the prelimbic cortex further demonstrated a significant main effect of the context (*F*_1,7_ = 24.41; *p* < 0.0001). The percentage of Fos-IR nuclei was higher in context A than in context B (*p* < 0.001).

### Experiment 4: Muscimol + Baclofen Inactivation of the Prelimbic Cortex Decreased the Context-Induced Reinstatement of Ethanol Seeking

In this experiment, we used a reversibly inactivating procedure by applying muscimol + baclofen (McFarland and Kalivas, 2001; Bossert et al., 2011; Bossert et al., 2012; Cruz et al., 2014) to determine the role of the prelimbic cortex in the context-induced reinstatement of ethanol seeking. The results of this experiment indicated a significant interaction among context (A, B), lever (active, inactive), and drug (vehicle, muscimol + baclofen), *F*_1,58_ = 5.15; *p* < 0.05.

**FIGURE 5:**
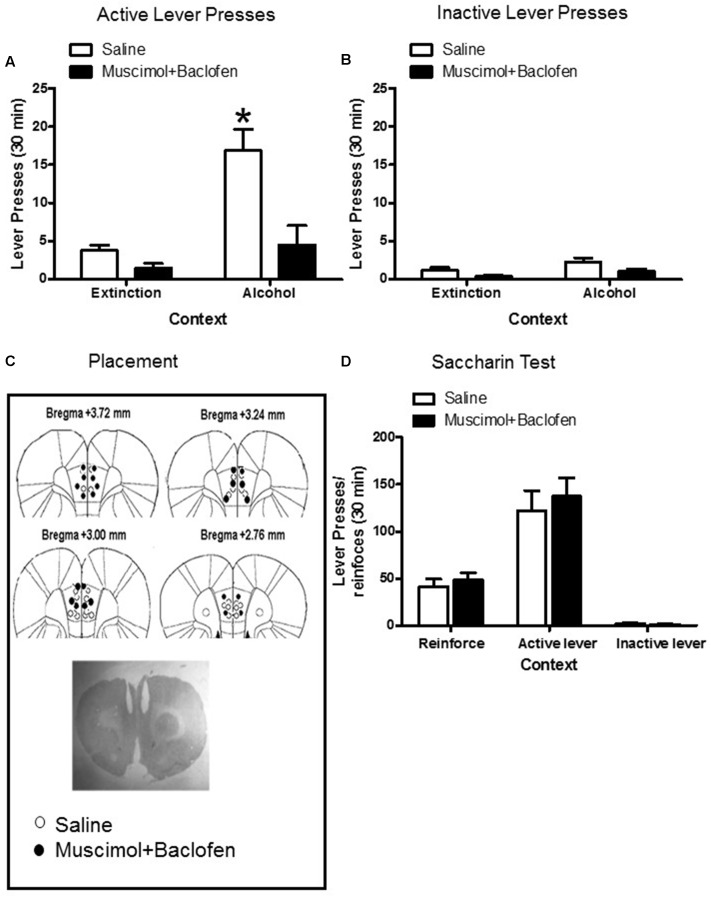
Muscimol + Baclofen inactivation of the prelimbic cortex decreased the context-induced reinstatement of ethanol self-administration. **(A)** Total active lever-presses during the context-induced reinstatement of ethanol seeking on the test day. **(B)** Total inactive lever-presses during the context-induced reinstatement of ethanol seeking on the test day (*n* = 6–9 per group). **(C)** Dots indicate the approximate area of the injector tip. **(D)** Local inactivation of the prelimbic cortex with muscimol+baclofen had no effect on high-rate 0.2% saccharin reinforcements responding (*n* = 8–10 per group). Data are depicted as mean ± standard error of the mean. ^∗^Different from extinction context B, *p* < 0.05.

For active lever, the two-way ANOVA indicated significant interaction between context (A, B) and drug (vehicle, muscimol + baclofen) factors, *F*_1,29_ = 5.50; *p* < 0.001. The *post hoc* statistical analysis indicated that the muscimol + baclofen injections into the prelimbic cortex attenuated the active lever pressing in context A (p < 0.001), but not in context B (**Figure 5A**).

For inactive lever, none interaction was observed between context (A, B) and drug (vehicle, muscimol + baclofen) factors, *F*_1,29_ = 0.20; *p* > 0.05 (**Figure 5B**).

Finally, to rule out the possibility that this effect was due to motor deficits, we re-trained 18 rats that previously participated in Experiment 4 to lever presses for a 0.2% saccharin solution. After a stable responding was observed, we determined the effect of muscimol + baclofen or vehicle injections into the prelimbic cortex on high-rate operant responding for saccharin. The local inactivation of the prelimbic cortex with muscimol + baclofen had no effect on high-rate saccharin responding, indicating that the observed muscimol + baclofen effects on the context-induced reinstatement of ethanol seeking were not due to motor deficits (Interaction [lever presses/reinforcements *versus* saline/baclofen]: *F*_1,47_ = 0.44; *p* = 0.51; **Figure 5D**).

## Discussion

We investigated the role of the prelimbic cortex in the context-induced reinstatement of ethanol seeking. In line with previous reports (Fuchs et al., 2005; Crombag et al., 2008), ethanol seeking was induced by re-exposure to the context A after the extinction of drug self-administration in a different context. The context-induced ethanol seeking increased the expression of the neural activity marker Fos in 7.7% of the prelimbic neurons *versus* 2.5% and 1.1% in the infralimbic and cingulate cortex, respectively. The reversible inactivation of the prelimbic cortex using the GABA agonists muscimol + baclofen attenuated the context-induced reinstatement of ethanol seeking.

Context-induced reinstatement of extinguished drug seeking has been observed with several drugs of abuse including speedball (a heroin-cocaine combination) (Crombag and Shaham, 2002), cocaine (Cruz et al., 2014), heroin (Bossert et al., 2012), nicotine (Diergaarde et al., 2008), and alcoholic beer (Willcocks and McNally, 2013). Our results also corroborated previous clinical studies indicating that a context previously associated with ethanol use often provokes relapse during abstinence (O’Brien et al., 1990; Kirk and de Wit, 2000; Litt and Cooney, 2000).

Our results also corroborate with earlier studies showing that the alcohol associated context induced reinstatement of alcohol self-administration (Marchant et al., 2013; Willcocks and McNally, 2013). However, in the present study, we used a different protocol. For instance, Willcocks and McNally (2013) used an alcoholic beer, while we used an ethanol diluted in water. Further, Marchant et al. (2013) used punishment (footshock) for inducing suppression of ethanol seeking and Cannella et al. (2016) used ethanol preferring instead of Long-Evans rats for showing the ability of context induces reinstatement of ethanol seeking. The use of an alcoholic beer requires an additional control group (non-alcoholic beer). Footshock-induced extinction of ethanol seeking is not a useful procedure for studying plasticity related to context-induced relapse, because footshock might cause its own plasticity. Thus, our procedure may be considered an easy, reliable, robust, and alternative model to explore the mechanisms of context-induced relapse of ethanol seeking.

Distinct brain regions from the medial cortex have been implicated in the extinction and reinstatement of drug seeking (Fuchs et al., 2005; Lasseter et al., 2010; Bossert et al., 2011; Willcocks and McNally, 2013; Marchant et al., 2015). Our findings demonstrated a critical role of the prelimbic cortex in the context-induced relapse to ethanol seeking, which is consistent with previous studies (Willcocks and McNally, 2013). Willcocks and McNally (2013) showed that the reversible inactivation of the dorsal medial cortex by muscimol + baclofen attenuated the context-induced reinstatement of alcoholic beer seeking. There is a body of evidence indicating a role of the prelimbic cortex in different forms of reinstatement of drug seeking (Lasseter et al., 2010; Shen et al., 2014; Marchant et al., 2015; Stefanik et al., 2016). For instance, McFarland and Kalivas (2001) demonstrated that the pharmacological inactivation of the prelimbic cortex attenuated the primed reinstatement of cocaine seeking. Furthermore, Fuchs et al. (2005) demonstrated that the inactivation of the prelimbic cortex prevented the cue-induced reinstatement of cocaine self-administration. Taken together, these studies suggested that the prelimbic cortex could be a common pathway for relapse.

Evidence has also implicated the prelimbic cortex in associative learning and retrieval of remote long-term memory (Quinn et al., 2008; Euston et al., 2012). In particular, studies have demonstrated that the prelimbic cortex receives information from the emotion-related structure that is important for learning and associative memory (Peters et al., 2009; Euston et al., 2012). Furthermore, the prelimbic cortex receives a broad range of sensory and limbic inputs from the hippocampus, amygdala, orbital frontal cortex, and ventral tegmental area, which can be activated by contextual cues (Miller, 2000a,b; Mulder et al., 2000; Miller and Cohen, 2001). Additionally, the activation of the medial cortex–nucleus accumbens core–ventral pallidum pathway has been implicated in context-induced reinstatement of drug seeking (Kalivas, 2008; Peters et al., 2008; Van den Oever et al., 2010). Furthermore, it was demonstrated that the activation of these inputs can lead to context-dependent outcomes (Miller and Cohen, 2001).

Moreover, the glutamatergic and GABAergic neurons in the prelimbic cortex receive stimuli-specific patterns of inputs from the cortex, amygdala, and ventral tegmental area, which play a critical role in cognitive and emotional regulation and memory consolidation (Brandstatter et al., 1995). For instance, the hippocampal projections landing cells in the prelimbic cortex provide an essential input by which the spatial information can be integrated into the cognitive process (Floresco et al., 1997). Based on these previous findings, we assume that the activation of the prelimbic cortex by contextual cues could increase drug-seeking behaviors and cause relapse to drug use (Marchant et al., 2015).

Learned associations are hypothesized to be encoded within sparsely distributed neuronal patterns, called neuronal ensembles (Pennartz et al., 1994, 2004; Guzowski et al., 2004; Buzsaki and Moser, 2013; Cruz et al., 2013, 2015). We found that the context-induced reinstatement of ethanol seeking correlated with Fos induction in approximately 7.7% of the neurons in the prelimbic cortex. Previous studies have demonstrated the involvement of the neuronal ensembles in context-induced reinstatement of drug seeking (Cruz et al., 2013, 2014, 2015; Leão et al., 2015; Rubio et al., 2015). In some of these studies, the drug-associated contexts increased the Fos expression in the cortex and nucleus accumbens, while the selective inactivation of these Fos-expressing neurons attenuated the drug seeking behavior when exposed again to the drug-associated contexts and cues on the test day (Bossert et al., 2011; Cruz et al., 2014). These data indicated that the ability of contexts to induce reinstatement of drug seeking is mediated by specific patterns of Fos-expressing neuronal ensembles that are selected by these contexts (Bossert et al., 2011; Cruz et al., 2013, 2014, 2015; Rubio et al., 2015; Warren et al., 2016). Thus, our study suggested that a small neuronal subset of the prelimbic cortex encodes the learned association between ethanol and the drug-associated context, and that the reactivation of this small neuronal subset may lead to the reinstatement of ethanol seeking behavior. However, more studies are necessary to demonstrate a causal role of the prelimbic neuronal ensembles in the context-induced reinstatement of ethanol seeking.

## Conclusion

We described a new rat model of context-induced relapse to ethanol and confirmed morphologically and functionally the role of the prelimbic cortex in the context-induced reinstatement of ethanol seeking. Additionally, we demonstrated that the context-induced reinstatement of ethanol seeking was correlated with the activation of a small subset of neurons in the prelimbic cortex.

## Author Contributions

PP, RL, PB, PdO, CP, and FC designed the behavioral and histochemistry experiments. PP, RL, PB, PdO, and FC performed the behavioral experiments, while PP, RL, PB, and FC performed the histochemistry experiments. RL, FC, PP, RL, PB, PdO, CP, and FC wrote the paper.

## Conflict of Interest Statement

The authors declare that the research was conducted in the absence of any commercial or financial relationships that could be construed as a potential conflict of interest.

## References

[B1] Becker J. B. Koob G. F. (2016). Sex differences in animal models: focus on addiction. *Pharmacol. Rev.* 68 242–263. 10.1124/pr.115.011163 26772794 PMC4813426

[B2] Bossert J. M. Marchant N. J. Calu D. J. Shaham Y. (2013). The reinstatement model of drug relapse: recent neurobiological findings, emerging research topics, and translational research. *Psychopharmacology* 229 453–476. 10.1007/s00213-013-3120-y 23685858 PMC3770775

[B3] Bossert J. M. Stern A. L. Theberge F. R. Cifani C. Koya E. Hope B. T. (2011). Ventral medial prefrontal cortex neuronal ensembles mediate context-induced relapse to heroin. *Nat. Neurosci.* 14 420–422. 10.1038/nn.2758 21336273 PMC3077927

[B4] Bossert J. M. Stern A. L. Theberge F. R. Marchant N. J. Wang H. L. Morales M. (2012). Role of projections from ventral medial prefrontal cortex to nucleus accumbens shell in context-induced reinstatement of heroin seeking. *J. Neurosci.* 32 4982–4991. 10.1523/JNEUROSCI.0005-12.2012 22492053 PMC3335169

[B5] Bouton M. E. (2011). Learning and the persistence of appetite: extinction and the motivation to eat and overeat. *Physiol. Behav.* 103 51–58. 10.1016/j.physbeh.2010.11.025 21134389

[B6] Bouton M. E. (2014). Why behavior change is difficult to sustain. *Prev. Med.* 68 29–36. 10.1016/j.ypmed.2014.06.010 24937649 PMC4287360

[B7] Bouton M. E. Garcia-Gutierrez A. Zilski J. Moody E. W. (2006). Extinction in multiple contexts does not necessarily make extinction less vulnerable to relapse. *Behav. Res. Ther.* 44 983–994. 10.1016/j.brat.2005.07.007 16198302

[B8] Bouton M. E. Schepers S. T. (2015). Renewal after the punishment of free operant behavior. *J. Exp. Psychol. Anim. Learn. Cogn.* 41 81–90. 10.1037/xan0000051 25706548 PMC4339226

[B9] Bouton M. E. Todd T. P. Leon S. P. (2014). Contextual control of discriminated operant behavior. *J. Exp. Psychol. Anim. Learn. Cogn.* 40 92–105. 10.1037/xan0000002 24000907 PMC4028427

[B10] Brandstatter J. H. Greferath U. Euler T. Wassle H. (1995). Co-stratification of GABAA receptors with the directionally selective circuitry of the rat retina. *Vis. Neurosci.* 12 345–358. 10.1017/S0952523800008026 7786855

[B11] Burattini C. Gill T. M. Aicardi G. Janak P. H. (2006). The ethanol self-administration context as a reinstatement cue: acute effects of naltrexone. *Neuroscience* 139 877–887. 10.1016/j.neuroscience.2006.01.009 16516392

[B12] Buzsaki G. Moser E. I. (2013). Memory, navigation and theta rhythm in the hippocampal-entorhinal system. *Nat. Neurosci.* 16 130–138. 10.1038/nn.3304 23354386 PMC4079500

[B13] Calu D. J. Kawa A. B. Marchant N. J. Navarre B. M. Henderson M. J. Chen B. (2013). Optogenetic inhibition of dorsal medial prefrontal cortex attenuates stress-induced reinstatement of palatable food seeking in female rats. *J. Neurosci.* 33 214–226. 10.1523/JNEUROSCI.2016-12.2013 23283335 PMC3711609

[B14] Cannella N. Kallupi M. Li H. W. Stopponi S. Cifani C. Ciccocioppo R. (2016). Neuropeptide S differently modulates alcohol-related behaviors in alcohol-preferring and non-preferring rats. *Psychopharmacology* 233 2915–2924. 10.1007/s00213-016-4333-7 27235017 PMC4935615

[B15] Crombag H. S. Bossert J. M. Koya E. Shaham Y. (2008). Review. Context-induced relapse to drug seeking: a review. *Philos. Trans. R. Soc. Lond. B Biol. Sci.* 363 3233–3243. 10.1098/rstb.2008.0090 18640922 PMC2607323

[B16] Crombag H. S. Shaham Y. (2002). Renewal of drug seeking by contextual cues after prolonged extinction in rats. *Behav. Neurosci.* 116 169–173. 10.1037/0735-7044.116.1.169 11895178

[B17] Cruz F. C. Babin K. R. Leao R. M. Goldart E. M. Bossert J. M. Shaham Y. (2014). Role of nucleus accumbens shell neuronal ensembles in context-induced reinstatement of cocaine-seeking. *J. Neurosci.* 34 7437–7446. 10.1523/JNEUROSCI.0238-14.2014 24872549 PMC4035511

[B18] Cruz F. C. Javier Rubio F. Hope B. T. (2015). Using c-fos to study neuronal ensembles in corticostriatal circuitry of addiction. *Brain Res.* 1628 157–173. 10.1016/j.brainres.2014.11.005 25446457 PMC4427550

[B19] Cruz F. C. Koya E. Guez-Barber D. H. Bossert J. M. Lupica C. R. Shaham Y. (2013). New technologies for examining the role of neuronal ensembles in drug addiction and fear. *Nat. Rev. Neurosci.* 14 743–754. 10.1038/nrn3597 24088811 PMC4530016

[B20] Diergaarde L. de Vries W. Raaso H. Schoffelmeer A. N. De Vries T. J. (2008). Contextual renewal of nicotine seeking in rats and its suppression by the cannabinoid-1 receptor antagonist Rimonabant (SR141716A). *Neuropharmacology* 55 712–716. 10.1016/j.neuropharm.2008.06.003 18588903

[B21] Euston D. R. Gruber A. J. McNaughton B. L. (2012). The role of medial prefrontal cortex in memory and decision making. *Neuron* 76 1057–1070. 10.1016/j.neuron.2012.12.002 23259943 PMC3562704

[B22] Floresco S. B. Seamans J. K. Phillips A. G. (1997). Selective roles for hippocampal, prefrontal cortical, and ventral striatal circuits in radial-arm maze tasks with or without a delay. *J. Neurosci.* 17 1880–1890. 9030646 10.1523/JNEUROSCI.17-05-01880.1997 PMC6573377

[B23] Fox H. C. Hong K. A. Sinha R. (2008). Difficulties in emotion regulation and impulse control in recently abstinent alcoholics compared with social drinkers. *Addict. Behav.* 33 388–394. 10.1016/j.addbeh.2007.10.002 18023295

[B24] Fuchs R. A. Evans K. A. Ledford C. C. Parker M. P. Case J. M. Mehta R. H. (2005). The role of the dorsomedial prefrontal cortex, basolateral amygdala, and dorsal hippocampus in contextual reinstatement of cocaine seeking in rats. *Neuropsychopharmacology* 30 296–309. 10.1038/sj.npp.1300579 15483559

[B25] Gauggel S. Heusinger A. Forkmann T. Boecker M. Lindenmeyer J. Cox W. M. (2010). Effects of alcohol cue exposure on response inhibition in detoxified alcohol-dependent patients. *Alcohol. Clin. Exp. Res.* 34 1584–1589. 10.1111/j.1530-0277.2010.01243.x 20586755

[B26] Guzowski J. F. Knierim J. J. Moser E. I. (2004). Ensemble dynamics of hippocampal regions CA3 and CA1. *Neuron* 44 581–584. 10.1016/j.neuron.2004.11.003 15541306

[B27] Ishikawa A. Ambroggi F. Nicola S. M. Fields H. L. (2008). Contributions of the amygdala and medial prefrontal cortex to incentive cue responding. *Neuroscience* 155 573–584. 10.1016/j.neuroscience.2008.06.037 18640246 PMC2900834

[B28] Kalivas P. W. (2008). Addiction as a pathology in prefrontal cortical regulation of corticostriatal habit circuitry. *Neurotox. Res.* 14 185–189. 10.1007/BF03033809 19073425

[B29] Kalivas P. W. McFarland K. (2003). Brain circuitry and the reinstatement of cocaine-seeking behavior. *Psychopharmacology* 168 44–56. 10.1007/s00213-003-1393-2 12652346

[B30] Kirk J. M. de Wit H. (2000). Individual differences in the priming effect of ethanol in social drinkers. *J. Stud. Alcohol.* 61 64–71. 10.15288/jsa.2000.61.64 10627098

[B31] Koya E. Uejima J. L. Wihbey K. A. Bossert J. M. Hope B. T. Shaham Y. (2009a). Role of ventral medial prefrontal cortex in incubation of cocaine craving. *Neuropharmacology* 56(Suppl. 1), 177–185. 10.1016/j.neuropharm.2008.04.022 18565549 PMC2635336

[B32] Lasseter H. C. Xie X. Ramirez D. R. Fuchs R. A. (2010). Prefrontal cortical regulation of drug seeking in animal models of drug relapse. *Curr. Top. Behav. Neurosci.* 3 101–117. 10.1007/7854_2009_19 21161751 PMC4381832

[B33] Leão R. M. Cruz F. C. Vendruscolo L. F. de Guglielmo G. Logrip M. L. Planeta C. S. (2015). Chronic nicotine activates stress/reward-related brain regions and facilitates the transition to compulsive alcohol drinking. *J. Neurosci.* 35 6241–6253. 10.1523/JNEUROSCI.3302-14.2015 25878294 PMC4397613

[B34] Li X. Zeric T. Kambhampati S. Bossert J. M. Shaham Y. (2015a). The central amygdala nucleus is critical for incubation of methamphetamine craving. *Neuropsychopharmacology* 40 1297–1306. 10.1038/npp.2014.320 25475163 PMC4367476

[B35] Litt M. D. Cooney N. L. (2000). Re: comments on “Reactivity to alcohol-related stimuli in the laboratory and in the field: predictors of craving in treated alcoholics: a reply”. *Addiction* 95 1107–1108. 10.1046/j.1360-0443.2000.957110715.x 10962778

[B36] Litt M. D. Cooney N. L. Morse P. (2000). Reactivity to alcohol-related stimuli in the laboratory and in the field: predictors of craving in treated alcoholics. *Addiction* 95 889–900. 10.1046/j.1360-0443.2000.9568896.x 10946438

[B37] Marchant N. J. Campbell E. J. Whitaker L. R. Harvey B. K. Kaganovsky K. Adhikary S. (2016). Role of ventral subiculum in context-induced relapse to alcohol seeking after punishment-imposed abstinence. *J. Neurosci.* 36 3281–3294. 10.1523/JNEUROSCI.4299-15.2016 26985037 PMC4792939

[B38] Marchant N. J. Kaganovsky K. Shaham Y. Bossert J. M. (2015). Role of corticostriatal circuits in context-induced reinstatement of drug seeking. *Brain Res.* 1628 219–232. 10.1016/j.brainres.2014.09.004 25199590 PMC4362860

[B39] Marchant N. J. Khuc T. N. Pickens C. L. Bonci A. Shaham Y. (2013). Context-induced relapse to alcohol seeking after punishment in a rat model. *Biol. Psychiatry* 73 256–262. 10.1016/j.biopsych.2012.07.007 22883434 PMC3517691

[B40] Marchant N. J. Rabei R. Kaganovsky K. Caprioli D. Bossert J. M. Bonci A. (2014). A critical role of lateral hypothalamus in context-induced relapse to alcohol seeking after punishment-imposed abstinence. *J. Neurosci.* 34 7447–7457. 10.1523/JNEUROSCI.0256-14.2014 24872550 PMC4035512

[B41] McCusker C. G. Brown K. (1990). Alcohol-predictive cues enhance tolerance to and precipitate “craving” for alcohol in social drinkers. *J. Stud. Alcohol.* 51 494–499. 10.15288/jsa.1990.51.494 2270057

[B42] McFarland K. Kalivas P. W. (2001). The circuitry mediating cocaine-induced reinstatement of drug-seeking behavior. *J. Neurosci.* 21 8655–8663. 11606653 10.1523/JNEUROSCI.21-21-08655.2001 PMC6762812

[B43] Miller E. K. (2000a). The prefrontal cortex and cognitive control. *Nat. Rev. Neurosci.* 1 59–65. 10.1038/35036228 11252769

[B44] Miller E. K. (2000b). The prefrontal cortex: no simple matter. *Neuroimage* 11 447–450. 10.1006/nimg.2000.0574 10806030

[B45] Miller E. K. Cohen J. D. (2001). An integrative theory of prefrontal cortex function. *Annu. Rev. Neurosci.* 24 167–202. 10.1146/annurev.neuro.24.1.167 11283309

[B46] Muigg P. Hetzenauer A. Hauer G. Hauschild M. Gaburro S. Frank E. (2008). Impaired extinction of learned fear in rats selectively bred for high anxiety–evidence of altered neuronal processing in prefrontal-amygdala pathways. *Eur. J. Neurosci.* 28 2299–2309. 10.1111/j.1460-9568.2008.06511.x 19019199 PMC2777258

[B47] Mulder A. B. Nordquist R. Orgut O. Pennartz C. M. (2000). Plasticity of neuronal firing in deep layers of the medial prefrontal cortex in rats engaged in operant conditioning. *Prog. Brain Res.* 126 287–301. 10.1016/S0079-6123(00)26020-2 11105653

[B48] O’Brien C. P. Childress A. R. McLellan T. Ehrman R. (1990). Integrating systemic cue exposure with standard treatment in recovering drug dependent patients. *Addict. Behav.* 15 355–365. 10.1016/0306-4603(90)90045-Y 2248109

[B49] Paxinos G. Watson C. (2005). *The Rat Brain in Stereotaxic Coordinates.* Amsterdam: Elsevier. 10.1016/0165-0270(80)90021-7 6110810

[B50] Pennartz C. M. Groenewegen H. J. Lopes da Silva F. H. (1994). The nucleus accumbens as a complex of functionally distinct neuronal ensembles: an integration of behavioural, electrophysiological and anatomical data. *Prog. Neurobiol.* 42 719–761. 10.1016/0301-0082(94)90025-6 7938546

[B51] Pennartz C. M. Lee E. Verheul J. Lipa P. Barnes C. A. McNaughton B. L. (2004). The ventral striatum in off-line processing: ensemble reactivation during sleep and modulation by hippocampal ripples. *J. Neurosci.* 24 6446–6456. 10.1523/JNEUROSCI.0575-04.2004 15269254 PMC6729862

[B52] Peters J. Kalivas P. W. Quirk G. J. (2009). Extinction circuits for fear and addiction overlap in prefrontal cortex. *Learn. Mem.* 16 279–288. 10.1101/lm.1041309 19380710 PMC4527308

[B53] Peters J. LaLumiere R. T. Kalivas P. W. (2008). Infralimbic prefrontal cortex is responsible for inhibiting cocaine seeking in extinguished rats. *J. Neurosci.* 28 6046–6053. 10.1523/JNEUROSCI.1045-08.2008 18524910 PMC2585361

[B54] Quinn J. J. Ma Q. D. Tinsley M. R. Koch C. Fanselow M. S. (2008). Inverse temporal contributions of the dorsal hippocampus and medial prefrontal cortex to the expression of long-term fear memories. *Learn. Mem.* 15 368–372. 10.1101/lm.813608 18441294 PMC3960031

[B55] Rhodes S. E. Killcross A. S. (2007). Lesions of rat infralimbic cortex enhance renewal of extinguished appetitive Pavlovian responding. *Eur. J. Neurosci.* 25 2498–2503. 10.1111/j.1460-9568.2007.05486.x 17445245

[B56] Rhodes S. E. Killcross S. (2004). Lesions of rat infralimbic cortex enhance recovery and reinstatement of an appetitive Pavlovian response. *Learn. Mem.* 11 611–616. 10.1101/lm.79704 15466316 PMC523080

[B57] Rocha A. Kalivas P. W. (2010). Role of the prefrontal cortex and nucleus accumbens in reinstating methamphetamine seeking. *Eur. J. Neurosci.* 31 903–909. 10.1111/j.1460-9568.2010.07134.x 20180839 PMC4346145

[B58] Rogers J. L. Ghee S. See R. E. (2008). The neural circuitry underlying reinstatement of heroin-seeking behavior in an animal model of relapse. *Neuroscience* 151 579–588. 10.1016/j.neuroscience.2007.10.012 18061358 PMC2238688

[B59] Rubio F. J. Liu Q. R. Li X. Cruz F. C. Leão R. M. Warren B. L. (2015). Context-induced reinstatement of methamphetamine seeking is associated with unique molecular alterations in Fos-expressing dorsolateral striatum neurons. *J. Neurosci.* 35 5625–5639. 10.1523/JNEUROSCI.4997-14.2015 25855177 PMC4388923

[B60] Shen H. W. Gipson C. D. Huits M. Kalivas P. W. (2014). Prelimbic cortex and ventral tegmental area modulate synaptic plasticity differentially in nucleus accumbens during cocaine-reinstated drug seeking. *Neuropsychopharmacology* 39 1169–1177. 10.1038/npp.2013.318 24232172 PMC3957111

[B61] Sinha R. (2009). Modeling stress and drug craving in the laboratory: implications for addiction treatment development. *Addict. Biol.* 14 84–98. 10.1111/j.1369-1600.2008.00134.x 18945295 PMC2734447

[B62] Stefanik M. T. Kupchik Y. M. Kalivas P. W. (2016). Optogenetic inhibition of cortical afferents in the nucleus accumbens simultaneously prevents cue-induced transient synaptic potentiation and cocaine-seeking behavior. *Brain Struct. Funt.* 221 1681–1689. 10.1007/s00429-015-0997-8 25663648 PMC5259736

[B63] UNODC (2016). *World Drug Report 2016.* New York, NY: United Nations 10.18356/603a2a94-en

[B64] Van den Oever M. C. Spijker S. Smit A. B. De Vries T. J. (2010). Prefrontal cortex plasticity mechanisms in drug seeking and relapse. *Neurosci. Biobehav. Rev.* 35 276–284. 10.1016/j.neubiorev.2009.11.016 19932711

[B65] Warren B. L. Mendoza M. P. Cruz F. C. Leao R. M. Caprioli D. Rubio F. J. (2016). Distinct fos-expressing neuronal ensembles in the ventromedial prefrontal cortex mediate food reward and extinction memories. *J. Neurosci.* 36 6691–6703. 10.1523/JNEUROSCI.0140-16.2016 27335401 PMC4916247

[B66] Willcocks A. L. McNally G. P. (2013). The role of medial prefrontal cortex in extinction and reinstatement of alcohol-seeking in rats. *Eur. J. Neurosci.* 37 259–268. 10.1111/ejn.12031 23106416

[B67] Willcocks A. L. McNally G. P. (2014). An extinction retrieval cue attenuates renewal but not reacquisition of alcohol seeking. *Behav. Neurosci.* 128 83–91. 10.1037/a0035595 24512068

